# Gaze-centered coding of proprioceptive reach targets after effector movement: Testing the impact of online information, time of movement, and target distance

**DOI:** 10.1371/journal.pone.0180782

**Published:** 2017-07-05

**Authors:** Stefanie Mueller, Katja Fiehler

**Affiliations:** Department of Experimental Psychology, Justus-Liebig University Giessen, Giessen, Hesse, Germany; University of Muenster, GERMANY

## Abstract

In previous research, we demonstrated that spatial coding of proprioceptive reach targets depends on the presence of an effector movement (Mueller & Fiehler, Neuropsychologia, 2014, 2016). In these studies, participants were asked to reach in darkness with their right hand to a proprioceptive target (tactile stimulation on the finger tip) while their gaze was varied. They either moved their left, stimulated hand towards a target location or kept it stationary at this location where they received a touch on the fingertip to which they reached with their right hand. When the stimulated hand was moved, reach errors varied as a function of gaze relative to target whereas reach errors were independent of gaze when the hand was kept stationary. The present study further examines whether (a) the availability of proprioceptive online information, i.e. reaching to an online versus a remembered target, (b) the time of the effector movement, i.e. before or after target presentation, or (c) the target distance from the body influences gaze-centered coding of proprioceptive reach targets. We found gaze-dependent reach errors in the conditions which included a movement of the stimulated hand irrespective of whether proprioceptive information was available online or remembered. This suggests that an effector movement leads to gaze-centered coding for both online and remembered proprioceptive reach targets. Moreover, moving the stimulated hand before or after target presentation did not affect gaze-dependent reach errors, thus, indicating a continuous spatial update of positional signals of the stimulated hand rather than the target location per se. However, reaching to a location close to the body rather than farther away (but still within reachable space) generally decreased the influence of a gaze-centered reference frame.

## Introduction

In everyday life, we naturally act on objects we experience by visual or somatosensory information, e.g. when grasping a seen object or scratching an itch on our arm. Goal-directed actions to a target in space require that the target and the effector performing the action (usually the hand) are coded in the same reference frame in order to compute a movement vector, i.e. the distance and direction the effector has to be displaced to reach the target. For reaching to visual targets, it has been consistently shown that humans use a gaze-centered reference frame in which the locations of target and effector are coded relative to gaze direction and updated after each gaze shift (for reviews see [1]; [2]; [3]). Gaze-centered coding has also been found for reaches to somatosensory targets even in absence of any visual information ([4]; [5]; [6]). However, this seems to be more variable depending on various factors (for a review see [7]), e.g. task demands ([8]) or effector movement ([9]; [10]; [11]).

In a previous study ([11]), we asked participants to reach to the location of a proprioceptive-tactile target, i.e. a touch on the fingertip of the left hand, while varying the presence of a movement of the left, stimulated hand in two conditions. In the moved condition, participants had to move their left hand forward, received a touch at one of the fingertips, and then moved their stimulated hand back before reaching to the remembered location in space where they had felt the touch. In the stationary condition, the touch was applied again to one of the fingertips of their left hand, but this time the stimulated hand remained stationary and participants had to reach to the location of the felt touch. We found that participants used a gaze-centered reference frame in the moved but not in the stationary condition suggesting that the movement of the stimulated hand increased the weighting of a gaze-centered reference frame. These findings may point to a superior spatial updating mechanism operating in gaze-centered coordinates for proprioceptive position signals in order to maintain spatial stability ([2]; [12]) despite movement.

In this study, the moved and the stationary conditions differed in the amount of proprioceptive online information about the target during reaching. While the location where the hand received the touch had to be remembered in external space in the moved condition, proprioceptive online information was available in the stationary condition. Regarding the two-streams-model of visual perception and action ([9]), it has been proposed that online information is used for actions and coded in an egocentric frame of reference (reference on the observer) whereas information that has to be retrieved from memory is coded in a more stable, allocentric (reference in the external world) reference frame ([13]). An analogue model has been suggested for the processing of somatosensory information ([14]). However, the notion of allocentric—and thus gaze-independent—coding for remembered reach targets has been questioned in the visual ([15], [16]) as well as in the somatosensory domain. For example, a study by Jones and Henriques ([4]) compared gaze-dependent reaching errors of remembered and online proprioceptive targets and found the same pattern of gaze-dependent reach errors in both conditions. Moreover, in our previous study ([11]), we even found evidence for gaze-centered (a subclass of egocentric reference frames) coding when reaching to remembered but not to online proprioceptive targets. However, this finding could be also explained by the availability of an effector movement which was present in the first but not in the latter condition.

In experiment 1, we aimed to disentangle the influence of movement from the influence of the available sensory information at the target location. We asked whether, beyond the movement of the stimulated hand, the availability of sensory information at the target location influences the spatial reference frame used for proprioceptive reaching. To this end, we conducted two conditions, a stationary-online and a moved-remembered condition, that resembled the conditions of our previous study ([11]) and compared these to a moved-online condition in which participants moved their left hand similar as in the moved-remembered condition but instead of reaching to the *remembered* location of the touch, they reached to the *current* location of the fingertip that had been touched. Thus, the new moved-online condition included a movement of the stimulated hand, similar as in the moved-remembered condition, and online proprioceptive information about the target during the reach, similar as in the stationary-online condition. Based on the results of our previous study, we expect a higher influence of gaze direction on reaching errors in the moved-remembered than in the stationary-online condition. Moreover, if the movement of the stimulated hand instead of the availability of proprioceptive information at the target location facilitates gaze-centered coding of proprioceptive reach targets, gaze-dependent errors in the moved-online condition should differ from the stationary-online condition but not from the moved-remembered condition. However, if the previously observed difference between the moved and the stationary condition was solely due to the difference in the availability of proprioceptive online information, reach errors in the moved-online condition should not vary as a function of gaze relative to target similar as in the stationary-online condition and different from the gaze-dependent pattern of reach errors in the moved-remembered condition. A partial influence of proprioceptive online information should be indicated by a reduced effect of gaze on reach errors in comparison to the moved condition.

As described above, the moved condition of our previous study ([11]) consisted of two movement phases: a movement towards the target location before the touch and a movement away from the target location after the touch. In experiment 2, we examined whether the use of a gaze-centered reference frame depended on the time of the effector movement, i.e. before versus after target presentation, and thus spatial updating of the target location and/or the stimulated hand. If the target position (touch on finger) requires a spatial update in a gaze-centered reference frame, we would expect a larger effect of gaze direction on the reach errors if the stimulated hand is moved after the target presentation (movement-after-target) than when it is moved before (movement-before-target). In contrast, if the position of the stimulated hand is constantly updated in space using a gaze-centered reference frame, we would expect a similar pattern of gaze-dependent reach errors in both conditions, movement before and after target presentation.

To clarify the results we obtained in experiment 1 and 2, we conducted experiment 3 in which we investigated the influence of target distance from the body on the use of a gaze-centered reference frame. More specifically, we aimed to distinguish between the influence of target distance and proprioceptive online information on gaze-centered coding of proprioceptive reach targets. To this end, we conducted a new, moved-online-far, condition and compared the results to the moved-remembered and the moved-online condition of experiment1 in which the targets were presented far and near from the participant, respectively. An influence of target distance would be indicated by a difference of gaze-dependent reach errors between the moved-online-far and moved-online (-near) condition whereas a difference of gaze-dependent reach errors between the moved-remembered and the moved-online-far condition would speak in favor of an influence of the availability of proprioceptive online information.

## Methods

### Participants

A total number of 20 healthy humans participated in this study. Three participants dropped out because they did not fulfill the criterion of maintaining fixation in at least 60% of the trials in each condition. All of the remaining 17 participants completed experiment 1 (8 female; age (mean ± standard deviation, SD): 24.35 ± 3.52 years, range: 19–31 years). A subset of this group also performed experiment 2 (12 participants; 5 female; age (mean ± SD): 24.00 ± 3.25 years, range: 19–31 years) and/or experiment 3 (11 participants; 6 female; age (mean ± SD): 24.00 ± 3.41 years, range: 20–29 years). All participants were right-handed as indicated by scores ≥ 83 (mean ± SD: 93 ± 8) on the German translation of the Edinburgh Handedness Inventory ([17]). Participants either received course credits or monetary compensation (8€/hour). The study was formally approved by the local ethics committee of the department of psychology and sports sciences of the Justus-Liebig University Giessen (Lokale Ethik-Kommission des Fachbereichs 06, LEK-FB06). Participants gave their written informed consent before participation.

### Apparatus

Participants were asked to reach with their right hand to a target that they had perceived on their left hand while their gaze direction was varied. In particular, the start location of the reaching hand, the gaze direction, and the target location were varied in the horizontal plane. Distances in visual degree are specified relative to straight ahead from the cyclopean eye with negative values denoting locations to the left and positive to the right.

**Fig 1:**
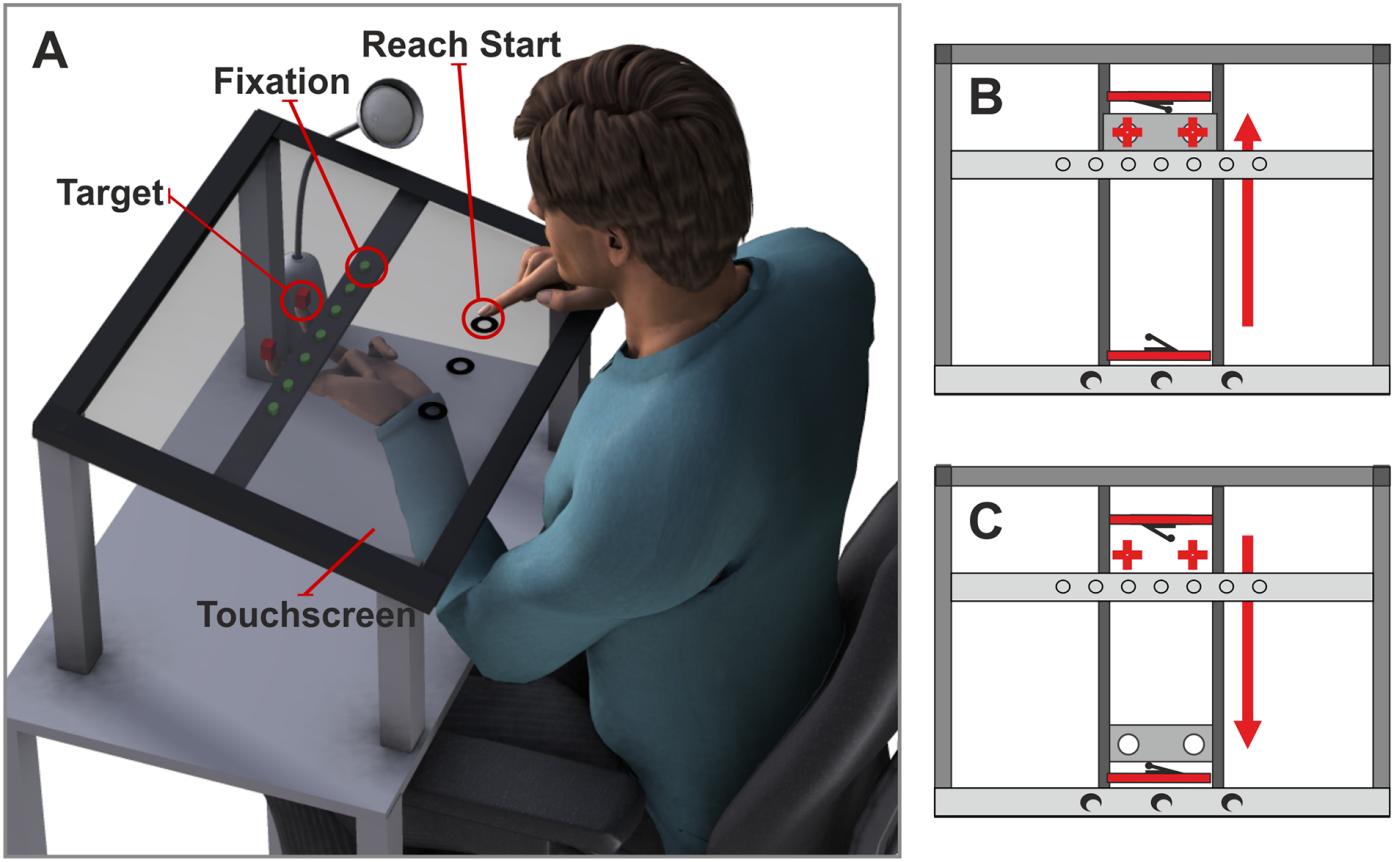
Schematic of the apparatus, side (A) and top view (B), (C). (A) Participants sat in front of the apparatus with their head fixed by a bite bar (not shown). They reached from one of three reach starts locations (black circles) to one of two targets (red cubes) while fixating one of seven LEDs (green circles). In the experiment, reach starts were marked by half-circled felt shapes attached on top of the horizontally mounted touch screen. The tactors (not shown) used to present mechanical touches and the LEDs used to vary gaze were each fixed in a metal plate under the touch screen. The index and ring finger of the left hand were kept in a slider (see (B)) that could be moved in the sagittal dimension and placed the fingers under the tactors. Black foil under the touch screen prevented seeing through the transparent touch screen. (B) and (C) Participants moved the slider between an endpoint near to (red rectangle at the bottom of the panel) and far from (red rectangle in the upper part) their body as shown here in a top view of the setup. Contact with the endpoints of a rail was signaled by mouse clicks. Panel (B) shows the slider at the position far from the body and panel (C) shows it at the position close to the body. In the moved-online-far condition, the tactors and LEDs were shifted to the near end of the touchscreen (not shown).

Participants sat in front of a table on which the apparatus was mounted (Fig 1A). Their head was fixed in a straight ahead posture by a personalized dental impression attached to a bite bar. The top layer of the apparatus consisted in a horizontally mounted touchscreen in a frame. The touchscreen (MagicTouch 2.0, Keytec, Inc., Garland, Texas, 43 x 33 x 0.3 cm) was used to record the reach endpoints with a resolution of 1680x1050 pixels. Reaches started at one of 3 reach starts at -10, 0, and +10° (spaced by 6.7 cm) with the central reach start at a distance of approximately 38 cm (27.5 cm in front of and 26 cm below) to the cyclopean eye. The reach starts were indicated by small (approximately the size of a fingertip) numbered cut-outs in a foil stripe covering the frontal side of the touchscreen.

Two thin metal plates were mounted under the touchscreen spanning from the left to the right side of the frame; one plate carried seven green LEDs (light emitting diodes, small circles in Fig 1) that were used to vary the gaze direction of the participant and the other plate carried two solenoids used for tactile stimulation.

The LEDs were spaced by approximately 5° (4 cm) with the central LED at a distance of 45 cm (37cm in front of and 26 cm below) to the cyclopean eye. The two solenoids were 1cm behind the LEDs (sagittal direction) with the center between the two solenoids at a distance of about 46 cm (39 cm in front of and 26 cm below) from the cyclopean eye. The distance between the two solenoids was set to 10° (8 cm). They were symmetrically arranged ± 5° relative to the cyclopean eye. When a current was applied to a solenoid, it pushed out a little pin in the downwards direction. The pin mechanically touched the tip of either the index or the ring finger of the participant’s left hand as it was located underneath the solenoids (touches are symbolized by red cubes Fig 1A or crosses Fig 1B and 1C).

The participants’ left arm rested on a platform under the touchscreen. They extended their left hand upwards and poked their index and ring finger through two holes in a slider. The slider could be moved in the sagittal direction (front to back) along two bars (Fig 1B and 1C). In the conditions in which the left hand had to be moved, participants were instructed to push/pull the slider till it reached the endpoint at the close or rear end of the bars (Fig 1B and 1C). One endpoint was located directly underneath the solenoids thus placing the fingers in the position to be touched by the pins (red crosses in Fig 1B and 1C). Black foil was attached to the underside of the touchscreen preventing the participants of seeing anything through the touchscreen except for lit LEDs. To avoid dark adaptation, a table lamp attached to the side of the apparatus was turned on between the trials until participants had placed their right index finger at the correct reach start. To conceal the sound of the solenoids, participants further wore in-ear headphones displaying white noise.

A head-mounted eye tracker (EyeLinkII eye tracker system, SR Research) recorded movements of the right eye with a sampling rate of 500 Hz. A horizontal 3-point calibration utilizing the LEDs at -10, 0, and 10° was performed before every condition. The eye data was analyzed offline to check the participants’ compliance with the instructions.

The stimuli presentation during the experiment was controlled by Presentation^®^ software (Version 17.2, www.neurobs.com); offline analyses were performed using MATLAB R2016a (TheMathWorks Inc., Natrick, MA) and SPSS 21 (SPSS Inc., Chicago, IL).

### General procedure

Participants reached with their right hand to a target location indicated by a mechanical touch on one of two fingertips of the left hand. Their gaze was varied by fixation lights that were turned off before reaching (but participants still fixated the location of the last fixation light) such that the reach was performed in total darkness. The same stimuli were presented in every condition (auditory cues, fixation light, and tactile stimulation) but conditions differed by whether and when participants performed a movement with their left stimulated hand and whether they reached to an online or a remembered location.

When the left hand had to be moved (a guided movement by pushing/pulling a slider along a rail, see red arrow in Fig 1B and 1C), the continuation of the trial procedure depended on the response of the participants as ensured by mouse clicks attached to the endpoints of the rail that signaled contact (red rectangles in Fig 1B and 1C). To keep the timing and trial duration similar between conditions, variable intervals accounting for the time of the movement were inserted into the conditions lacking movement in the respective trial phases.

**Fig 2:**
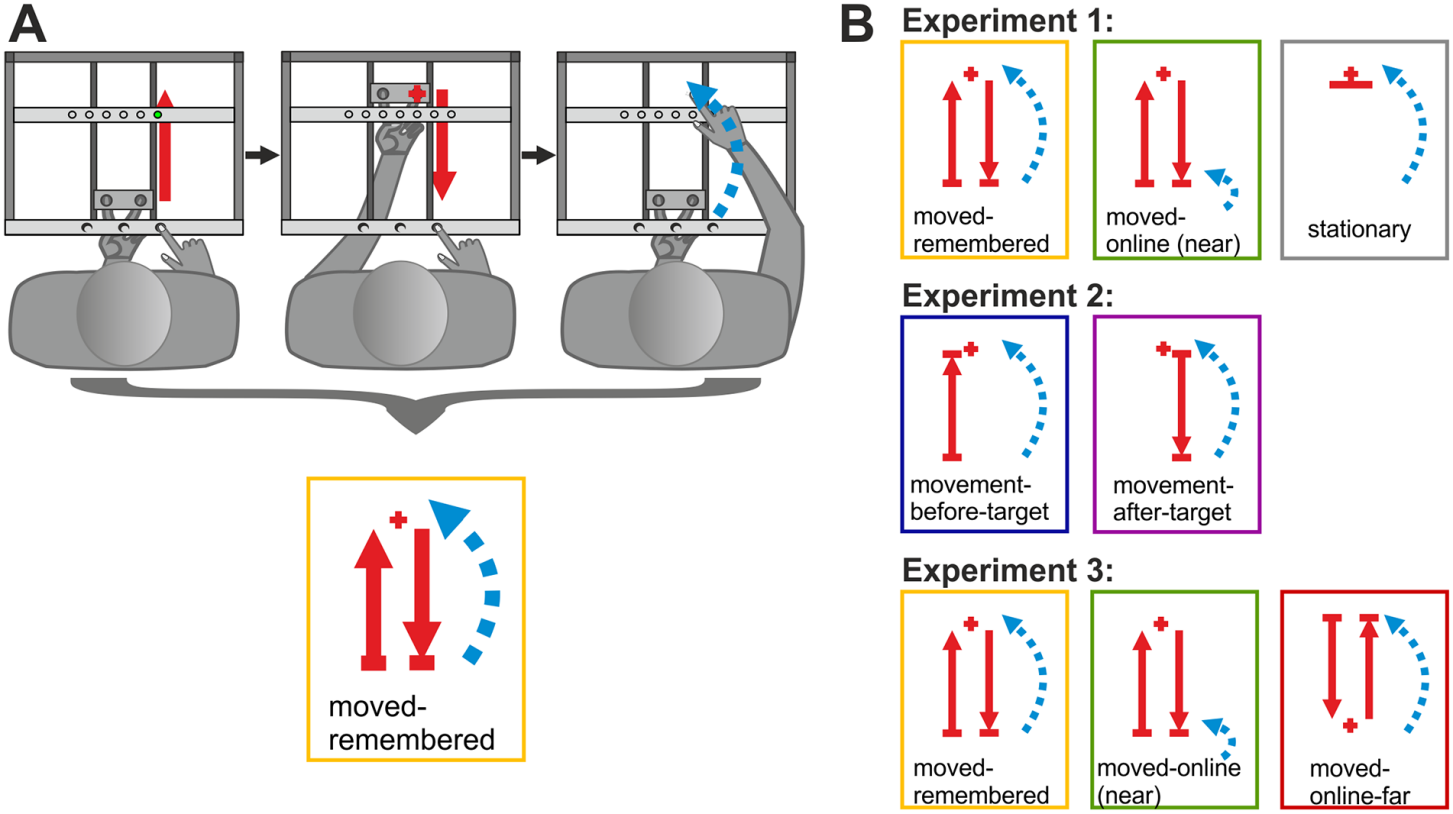
Schematic trial procedure of the moved-remembered condition (A) and symbolized summaries of conditions (B). (A) Every condition started with the announcement of the reach start while the table light was turned on. After participants had placed their right hand at the correct location, the table lamp turned off and a fixation LED turned on (green circle) prompting participants to fixate that location. The index and ring finger of the left hand were fixed in a slider under the touchscreen. The next trial phases differed between the conditions. In the moved-remembered condition as exemplified here, participants had to push their left hand forward (symbolized by red arrow) to the endpoint of the rail so that their fingertips were located directly under the tactors (1st panel). When the left hand arrived, the LED was extinguished and a touch (red cross) was applied to one of the fingertips. Then, the slider had to be pulled back (red arrow, 2nd panel). When the slider made contact with the near end of the rail, a go-signal prompted participants to reach (dashed blue arrow) to the target location while their gaze was still varied (3rd panel). The bottom panel shows the symbolized summary of the trial procedure: Red arrows show movement(s) of the left hand, the blue arrow symbolizes the reach of the right hand (with its tip pointing to the target location), red crosses symbolize the (coarse) location where the touch was presented. (B) Each row shows the symbolized summaries of conditions for one experiment. Red arrows symbolize movements of the left hand; dashed blue arrows symbolize reaches of the right hand. Red crosses indicate where the participant received the touch while the tip of the blue arrow (= reach) points to the target location. Note that the first two panels in Exp3 are the same as in Exp1.

The specific procedures within the single conditions are described in the following sections by first providing a detailed description for the stationary-online condition and then stating the differences for the succeeding conditions. Fig 2A illustrates a schematic of the trial procedure exemplified by the moved-remembered condition and shows a symbolized summary of each condition (Fig 2B).

### Experiment 1

In experiment 1 (Fig 2B, first row), we investigated whether the use of a gaze-centered reference frame that has been observed when an effector movement occurred before reaching to a proprioceptive target is further influenced by the availability of proprioceptive online information about the target location.

#### Stationary-online condition

In the stationary-online condition, participants did not move the left (target) hand but kept it at the same location directly under the solenoids throughout the condition. Thus, the location where the touch was delivered in space and the current location of the fingertip that had been touched coincided.

The trial started with the table lamp turned on. A computer-generated voice announced the number of the reach start (1, 2, or 3) on which the participant had to place the index finger of the right hand. When the finger was at the correct location, the table lamp was turned off and the computer-generated voice cued the participant to keep the finger at this position (“Stay!”) and to fixate the LED (“Fixate!”) that was turned on at this moment. The fixation light appeared for a randomized duration between 1400 and 1900 ms. This interval was chosen to account for the time of the left hand movement in the other conditions. After the fixation light was turned off, a short tactile touch of 50ms was presented on one of two fingertips of the left stimulated hand. The touch indicated the target location.

Between 800 and 1400 ms after the touch (random interval to again account for a left hand movement in other conditions), an auditory go-cue (“Go!”) prompted participants to reach with their right hand to the touch screen above the target location. The touch screen also prevented tactile feedback of the two hands. Successfully recorded reach endpoints were confirmed by a tone. The computer-generated voice then cued participants to start the next trial by pressing a button (“Next!”). When participants pressed the button, the table lamp was turned on and the next trial began.

#### Moved-remembered condition

In the moved-remembered condition, participants underwent the same trial procedure as in the previously described (and in all other) condition(s) but also moved their left hand as follows. The starting position of the left hand was at the endpoint of the rail that was close to the participant. When the fixation light was turned on, participants moved their left hand in the slider forward (away from the body) until the endpoint of the rail. Contact of the slider with the endpoint of the rail triggered that the fixation LED was turned off and that the target touch was applied to one of the two fingertips. Then, participants had to draw their left hand back (towards the body) but remember the location *where they had been touched in space*. The go-cue was presented after the slider contacted the respective endpoint of the rail (close to the body) and cued participants to reach to the remembered target location. Fig 2A provides a schematic of the trial procedure in the moved-remembered condition and illustrates how it translates into a symbolized summary of that condition.

#### Moved-online condition

In the moved-online condition, the trial procedure was exactly the same as in the moved-remembered condition but instead of reaching to the location in space where participants had been touched, they reached to the *finger* that had been touched. Since the left hand was moved after it had been touched, the current location of the finger was different from the location in space where the touch occurred.

### Experiment 2

In experiment 2 (Fig 2B, second row), we explored whether gaze-centered coding of proprioceptive reach targets depend on the time of movement, i.e. whether the movement of the stimulated hand occurred before or after the presentation of the target (touch on finger).

#### Movement-before-target condition

In the movement-before target condition, participants started each trial with their left hand at the endpoint close to their body. The trial procedure until the presentation of the fixation LED was the same as in the other conditions. While the fixation light was illuminated, participants moved their left hand in the slider forward (away from the body). After the left hand arrived at the endpoint under the solenoids (far from the body), a touch stimulated one of the two fingertips. The left, stimulated hand stayed at the location under the solenoids and when the go signal was presented after an interval of 800 to 1400 ms participants reached to the location where they had received the touch, i.e. to the current location of the target finger. The trial ended after the reach had been performed. Then, the left hand had to be moved back to the endpoint close to the body before the next trial began.

#### Movement-after-target condition

In the movement-after-target condition, participants started with their left hand at the endpoint under the solenoids (far from the body). After participants placed the index finger at the correct reach start (as described in detail for the stationary-online condition), the fixation LED was turned on for a variable interval of 1400 to 1900 ms. Afterwards, the touch was presented on one of the two fingertips. Participants had to remember the location in space. Then, they had to draw their left hand to the endpoint close to their body. After they arrived there, the go-signal was presented and cued them to reach to the location in space where they had received the touch. After the reach had been performed, the left hand had to be moved back to the endpoint far from the body before the next trial began.

### Experiment 3 –specific procedures

Experiment 3 (Fig 2B, third row) was conducted to clarify the results of experiment 1 and 2. In this experiment, the moved-online condition was designed in order to be comparable in terms of the left hand movement with the moved-remembered condition and in terms of the proprioceptive online information about the target location with the stationary-online condition. That means, participants performed the same movement as in the moved-remembered condition but reached to the current location of their touched fingertip. However, this resulted in a shorter target distance relative to the body than in the other conditions. Taken together, the results of experiment 1 and 2 raised the question whether the target distance from the body rather than the proprioceptive online information influences gaze-centered coding of proprioceptive reach targets. We post-hoc conducted a new condition, the moved-online-far condition, in which participants also moved their left hand to the target location, received proprioceptive online information about the target and reached for a similar, i.e. larger, distance. We slightly adjusted the apparatus by shifting the plates carrying the solenoids and the LEDs to the front end of the touchscreen so that the touches were now delivered at the endpoint of the movement close to the body. The moved-online-far condition was then compared with the moved-remembered and the moved-online condition of experiment 1.

#### Moved-online-far condition

Participants started with their left hand at the endpoint far from the body and had to draw their hand to the endpoint close to the body. Afterwards, they received the touch on their fingertip and then pushed their hand back to the endpoint far from their body. When contact was made with this endpoint, the go-cue was triggered and participants reached with their right hand to the current location of the fingertip that had been touched (= online). Thus, they had online proprioceptive feedback about the target location but in contrast to the moved-online condition of experiment 1, the target distance was comparable to all other conditions since the final position of the target was now at the endpoint far from the body.

### Design and data analyses

We recorded 2-dimensional (horizontal and sagittal) reach endpoints with a touchscreen that was mounted closely above the somatosensory targets. We varied gaze direction relative to the target locations (the retinal error or retinal eccentricity, RE) and analyzed whether gaze direction exerted an influence on reaching endpoints. The RE is calculated by subtracting the target position from the fixation position. Gaze was aligned with the target (at 0°) or -10°, -5°, 5°, or 10° relative to the target. The reaching hand started either 10° left or right of the target. One block consisted of all these combinations performed once, yielding 20 trials (2 targets x 5 fixations x 2 starts). In order to keep the upcoming target of a trial unpredictable after the start and the fixation location had been presented, we further included 4 catch trials per block in which the distance between the start and target as well as the fixation and the target location exceeded 10° (e.g. target at -5°, fixation at 10°, start at 10°). Catch trials were not analyzed. Every condition consisted of 5 blocks á 24 trials, thus 120 trials in total which took approximately 20 min to complete. In very few trials (<10 across all conditions and participants), a condition was interrupted due to technical reasons (e.g. the pin of a solenoid fell out and had to be reattached). In those cases, the current block was started again and completed together with the remaining blocks. Data of incomplete blocks were additionally included in the analyses.

The conditions were performed block wise in an almost counterbalanced order within experiment 1 (one more participant would have been needed for full counterbalancing) and a fully counterbalanced order within experiment 2. The new condition of experiment 3 was conducted post-hoc, thus always last, and compared with the data of the moved-remembered and moved-online condition obtained in experiment 1. Experiment 1 and 2 as well as the new condition of experiment 3 were conducted on different days.

The eye data was exported to a custom-written graphical user interface (GUI, programmed in MATLAB) and semi-automatically checked to ensure that participants followed the instruction to fixate the location of the LED until the reach was completed. More specifically, the GUI displayed the position of the right eye and the position of the presented stimuli (fixation and target locations) over time. We visually inspected if gaze achieved a stable position when the fixation light was displayed and if it remained within a range of ± 2.5° around this position until the end of the reach. If gaze exceeded the specified range, the trial was manually marked as invalid and later discarded from further analyses. The data was then exported into SPSS for further analyses. Participants who achieved less than 60% valid trials in any condition were excluded from the sample. Based on this criterion, 3 participants were excluded from the original sample of 20.

The mean and standard deviation of reach endpoints was calculated for every participant, condition, target, and retinal error (RE). Reach endpoints deviating from the mean by ± 2.5 SD were regarded as outliers and discarded. This was done separately in the horizontal and the sagittal dimension.

Without catch trials, we collected a total of 8737 trials (100%). After checking for correct fixations, 8060 trials (92.25%) remained in the sample. The outlier correction in the horizontal and the sagittal dimension further reduced the sample to the 8004 valid trials (91.61%) on which the following analyses were based.

### Statistical analyses

#### Analyses of reach endpoints

To check whether participants were able to distinguish the two target locations, one-factorial repeated-measure analyses of variance (RM-ANOVA) with the factor *target location* (-5°/+5°) were performed on the horizontal reach endpoints within every condition.

**Fig 3:**
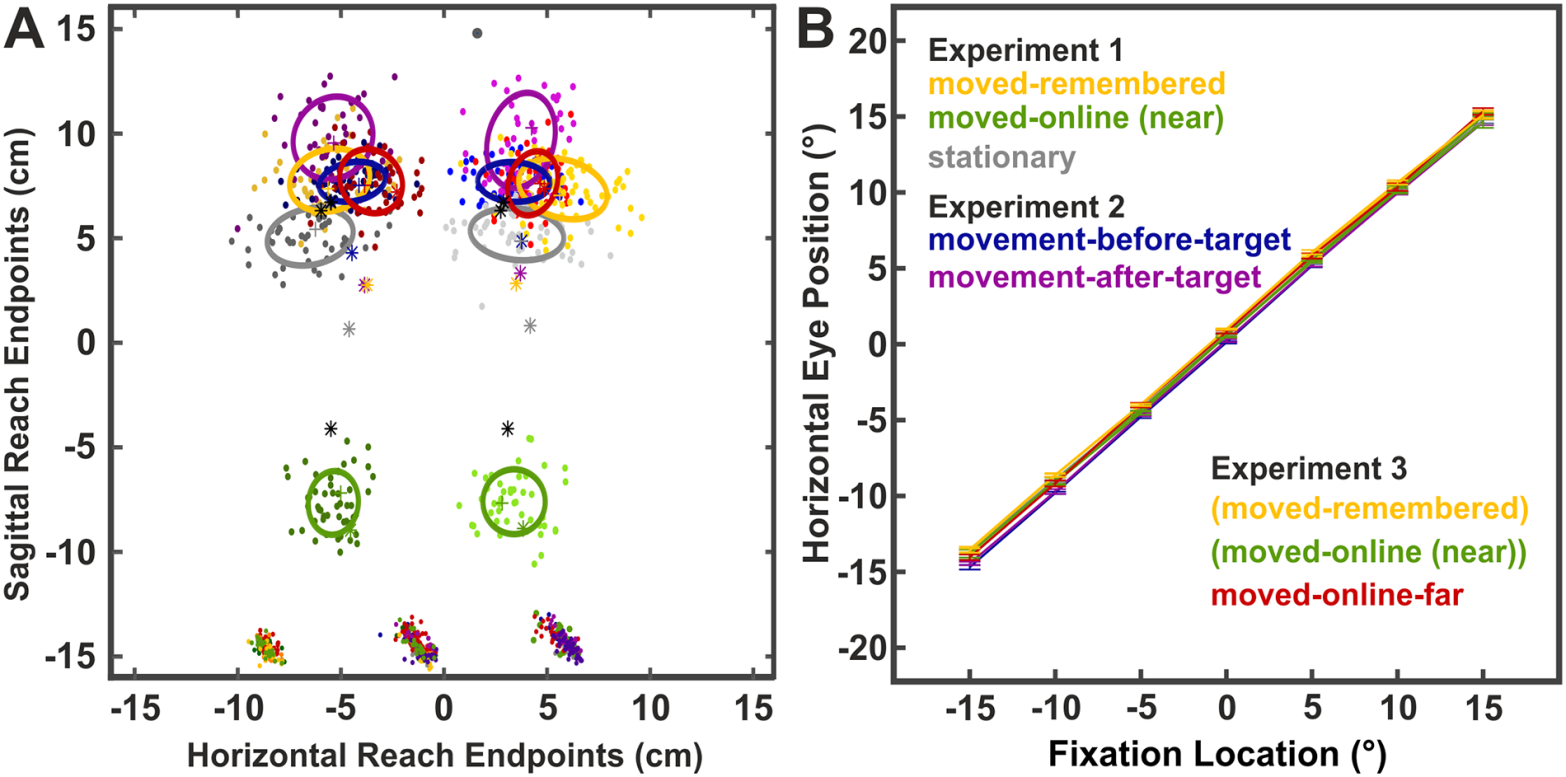
2D Reach endpoints and 50%-error ellipses of a single participant (A) and mean horizontal eye positions across participants (B) in all conditions. Colors indicate the conditions. (A) Colored ‘+’ symbols indicate the reach endpoints when gaze and target were aligned (RE = 0) for the exemplary participant; colored asterisks show the respective data averaged across all participants. Black asterisks indicate the physical target locations as obtained in a calibration procedure. Since the apparatus had to be changed for the moved-online-far condition, the physical target locations slightly differ from the other conditions (irrelevant for the analyses). In the moved-online (near) condition, the physical target locations were closer to the body of the participant (lower black asterisks). Colors of reach endpoints (dots) correspond to the respective ellipses, reaches to the right target are shown in slightly brighter colors than reaches to the left target. (B) Mean horizontal eye positions during the time of the touch response. Error bars display within-subjects standard errors.

Furthermore, we fitted ellipses (50% confidence interval) to the reach endpoints of every participant in each condition (see Fig 3). The areas of the ellipses were used as a measure for precision with large ellipses corresponding to low precision and vice versa. The ellipse areas were then analyzed by two-factorial RM-ANOVA (*condition x target location*) within each of the three experiments. The conditions vary within each experiment and are listed in the respective result section.

#### Analyses of gaze-dependent (horizontal) reach errors

We investigated how the influence of gaze direction on proprioceptive reaching changed depending on the availability of proprioceptive online information (experiment 1), the time of the effector movement (experiment 2), and the target distance from the observer (experiment3). We varied gaze relative to target in the horizontal plane and thus, only analyzed horizontal reach errors. To compare the influence of gaze across different target locations and independent of individual biases, we calculated the reach errors as a difference measure. More specifically, the mean reach endpoints of trials in which gaze and target were aligned were calculated within every participant, condition, and target location. Then, those mean reach endpoints of trials with a retinal error of zero (RE = 0) were subtracted from all trials where gaze and target location differed. Consequently, differences between reach errors reflect the influence of gaze direction on reaching independent of individual biases and target locations.

**Fig 4:**
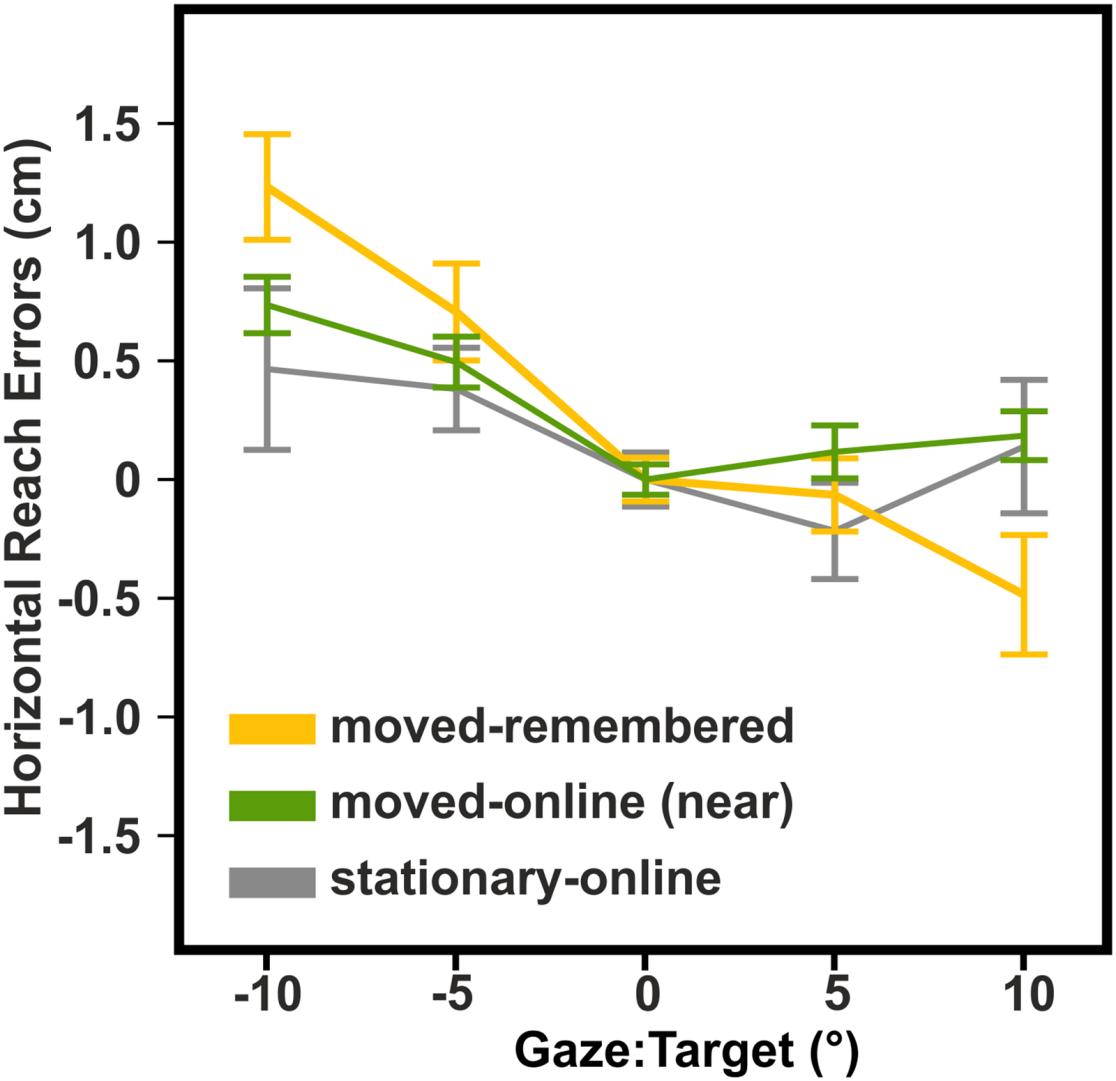
Exp1: Means and within-subjects standard errors of horizontal reach errors. Reach errors varied as a function of gaze relative to target in the moved-remembered and moved-online condition but not in the stationary-online condition. However, reach errors in the moved-remembered and moved-online condition differed significantly from another.

**Fig 5:**
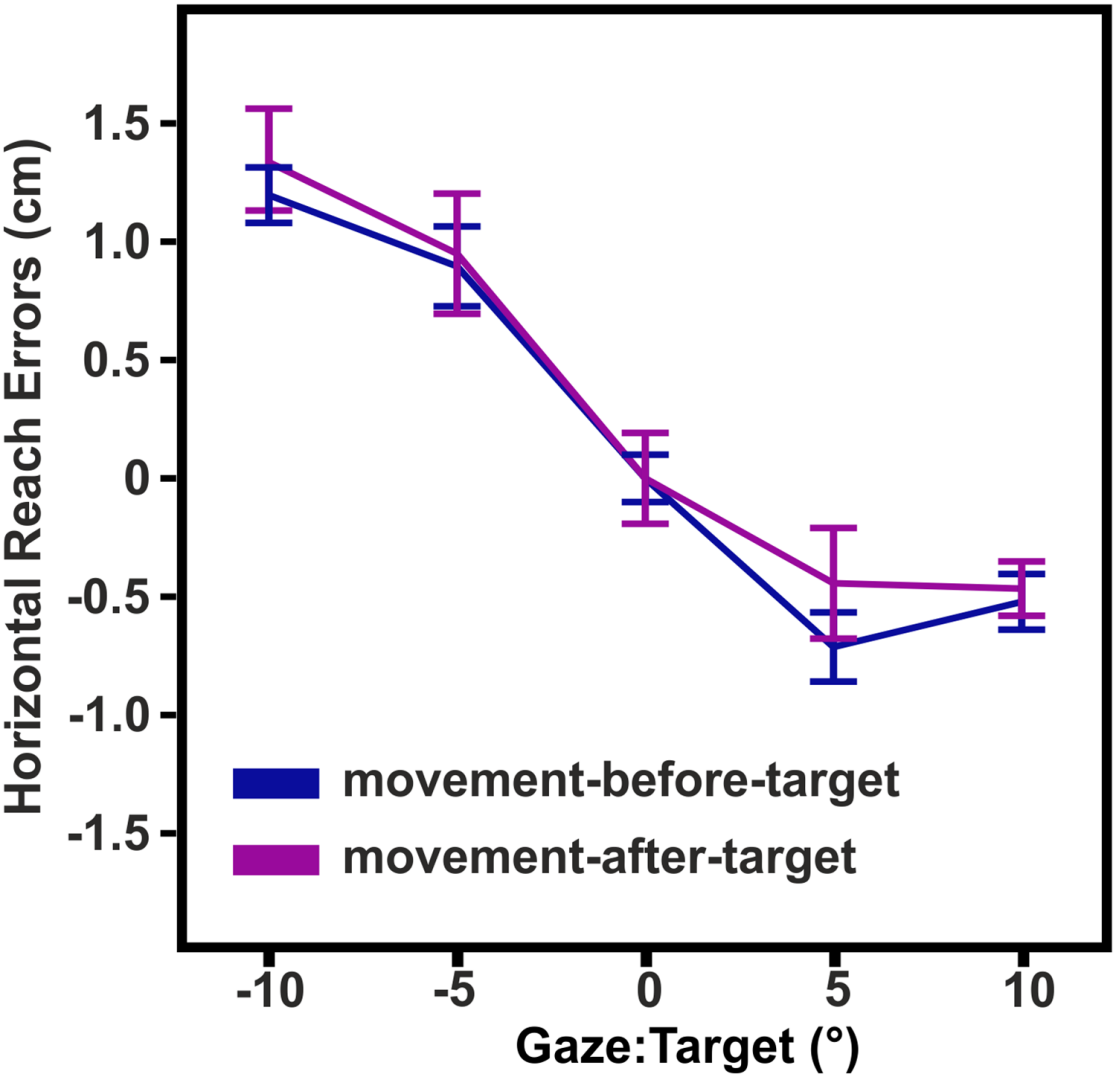
Exp2: Means and within-subjects standard errors of horizontal reach errors. Reach errors varied similar as a function of gaze relative to target between the movement-before-target and movement-after-target condition.

**Fig 6:**
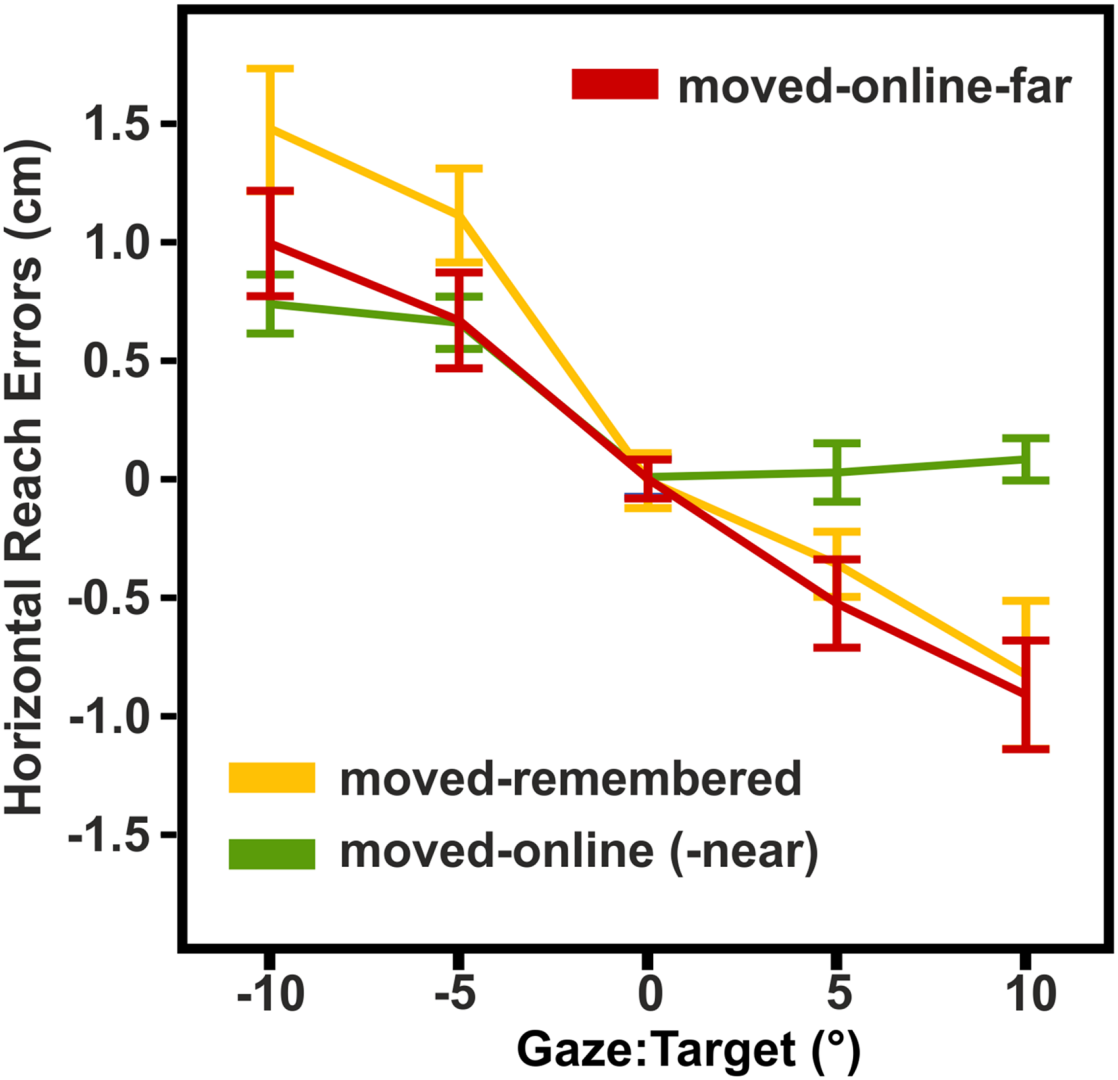
Exp3: Means and within-subjects standard errors of horizontal reach errors. Reach errors varied similar as a function of gaze relative to target in the moved-online-far and moved-online (-near) condition suggesting that the shorter target distance decreased the gaze-dependent effects in the moved-online (-near) condition.

The reach errors were statistically compared via two-factorial RM-ANOVA (*condition* x *retinal error*) in order to examine the influence of gaze-centered coding between the conditions within experiment 1 and experiment 2. The retinal error RE = 0 was excluded from the ANOVA since the conditions were normalized by this value and thus, it was zero for all participants and conditions. For illustrational purposes, it is shown as a reference in the figures (Figs 4–6). Significant interactions were further explored with one-factorial RM-ANOVA (*retinal error*). Within-subjects standard errors were calculated as described by Cousineau ([18]).

In addition, we regressed reach errors against retinal eccentricity and compared the resulting slopes between conditions. More specifically, a simple linear regression (reach error ~ constant + slope*RE) was fitted to the data of every participant in each condition and the slopes were compared by using one-factorial RM-ANOVA (*condition*) within experiment 1 and within experiment 2.

Analyses were performed at an α-level of 0.05. For post-hoc analyses, the α-level was corrected for multiple comparisons according to Bonferroni. When sphericity was violated as determined by the Mauchly’s test, Huynh-Feldt corrected p-values are reported.

The results of experiment 3 are reported without using inference statistics because two thirds of the data (the moved-remembered and moved-online-near condition) were already analyzed in experiment 1 and thus the experiment can be considered as exploratory.

## Results

### Participants were able to distinguish the target locations in every condition

First, we examined whether participants were able to distinguish the two target locations in every condition. The one-factorial RM-ANOVA on horizontal reach endpoints yielded a significant main effect of target location in every condition (F’s > 240.94, p’s < .001), thus confirming that participants detected the target and were able to discriminate the two locations.

### Precision of 2-dimensional reach endpoints

Ellipses with a 50% confidence interval were fitted to the reach endpoints of every participant in each condition. The areas of the ellipses were then analyzed via two-factorial RM-ANOVA (*condition x target location*) to compare the precision of reach endpoints between conditions and target locations within the 3 experiments. Fig 3 exemplarily shows the reach endpoints and ellipses of one participant in all conditions.

In experiment 1, we found a significant main effect of condition (moved-remembered, stationary-online, moved-online) and target (main effect condition: F_2,32_ = 16.73, p < .001; main effect target: F_1,16_ = 7.43, p = .015). The main effect of condition was further explored by paired t-tests comparing the precision between conditions averaged across target locations. Reach endpoints of the moved-online condition were significantly more precise than in the moved-remembered and stationary-online condition (moved-online vs. moved-remembered: t = -4.56, p < .001; moved-online vs. stationary: t = -5.42, p < .001) while the moved-remembered and stationary-online condition did not differ (t = 1.62, p = .125). To further explore the main effect of target location, we averaged the precision across conditions and compared the mean values between the two target locations. The result showed that reaches to the left target were more precise than reaches to the right target (t = -2.73, p = .015).

In experiment 2, the precision of reach endpoints was similar between the target locations and conditions (movement-before-target and movement-after-target condition) as indicated by the lack of significant main effects or interactions (F’s < 4.5, p’s > .057).

The analysis of experiment 3 (moved-online-far, moved-remembered, and moved-online condition) revealed significant main effects of condition (F_2,20_ = 11.93, p = .003) and target location (F_1,10_ = 7.54, p = .021). Further exploring the effect of condition, we found that the reach endpoints were most precise in the moved-online condition (moved-online vs. moved-remembered: t = -4.56; p < .001), followed by the moved-online-far condition (moved-online vs. moved-online -far: t = -3.36, p < .007), and the moved-remembered condition (moved-remembered vs. moved-online-far: t = -1.93; p = .014). The main effect of target location did not reach significance when we compared the ellipse areas between the two target locations averaged across conditions (t = -1.93; p = .072).

### Analyses of gaze-dependent reach errors and slopes

#### Exp1: The effect of the availability of online proprioceptive information and movement on gaze-centered coding

In previous studies ([19]; [10]), we observed that somatosensory reach targets are coded in a gaze-centered reference frame if a movement was performed before reaching. In experiment 1, we explored whether this effect is also influenced by the availability of proprioceptive online information about the target location.

To this end, we conducted 1) a condition in which the stimulated hand was stationary and online proprioceptive feedback about the target was available while the reach was performed (stationary-online condition), 2) a condition in which participants moved their left hand and had to remember the target location in space (moved-remembered condition), and 3) a condition in which participants moved their left hand and had online proprioceptive information about the target (moved-online condition). Fig 4 displays the results of experiment 1.

We compared how the reach errors varied as a function of gaze relative to target between the three conditions by calculating a two-factorial RM-ANOVA (*condition*: *stationary-online*, *moved-remembered*, *moved-online (3)* x *retinal error*: *-10*, *-5*, *5*, *10 (4)*). The analysis yielded a significant interaction of condition and retinal error (F_6,96_ = 2.92, p = .044). We further explored this interaction by calculating one-factorial RM-ANOVA (*retinal error (4)*) within each condition. The results showed that gaze direction exerted a significant influence on reach errors in the conditions where the left hand was moved before the reach (moved-remembered: F_3,48_ = 10.13, p = .002; moved-online: F_3,48_ = 5.22, p = .003) but not in the stationary-online condition (F_3,48_ = 1.06, p = .328). This result replicates the finding from our previous studies ([19]; [10]) that movement of the stimulated hand before reaching leads to gaze-centered coding of proprioceptive reach targets.

However, the availability of online proprioceptive information may have also influenced the spatial coding scheme such that the lack of proprioceptive online information rather than the movement per se led to the use of a gaze-centered reference frame for the encoding of memorized locations of proprioceptive reach targets. To investigate whether gaze-dependent reach errors vary depending on proprioceptive online information, we calculated a two-factorial RM-ANOVA to distinguish between the two conditions that included movement before reaching and that had yielded significant effects of gaze direction (*conditions*: *moved-remembered*, *moved-online x retinal error*). We found a significant interaction (F_3,48_ = 4.72, p = .024; α-level corrected for 2 comparisons p = .025) indicating a stronger influence of gaze direction on reach errors in the moved-remembered condition than in the moved-online condition. This suggests that the absence of proprioceptive online information does not solely account for the observed gaze-centered coding in the moved-conditions but that the proprioceptive online information seems to have a mitigating effect.

The analyses of the slopes support the analyses of the reach errors although effects were slightly reduced. The one-factorial RM-ANOVA (*condition*: *stationary-online*, *moved-remembered*, *moved-online*) comparing slopes between conditions did yield a statistical trend (F_2,32_ = 3.18, p = .055). Similar as for the reach errors, the slope was significantly different from zero in the moved-remembered (t = -3.40, p = .004) and moved-online condition (t = -3.00, p = .009) but not in the stationary-online condition (t = -0.82, p = .423). A paired t-test contrasting the slopes of the two conditions that included a movement before reaching (*moved-online* versus *moved-remembered*) yielded a significant difference (t = 2.47, p = .025) analogues to the analyses on reach errors.

#### Exp2: No differential influence of moving the left hand before or after the somatosensory target presentation

In previous studies ([19]; [10]) as well as in experiment 1 of the present study (moved-remembered condition), participants moved their left hand to the target location, received a touch, and moved this hand back before reaching to the location of the touch. With experiment 2, we addressed the question whether the time of movement, *before* versus *after* receiving the touch, influences gaze-centered coding of proprioceptive reach targets. A two-factorial RM-ANOVA (*conditions*: *movement-before-target*, *movement-after-target x retinal error*) was conducted to compare how reach errors varied as a function of gaze relative to target between the two conditions. The analysis yielded a significant main effect of *retinal error* (F_3,33_ = 21.76, p < .001) but no effects for condition (F_3,33_ = 0.43, p = .528) suggesting that both movements of the left hand—before and after target presentation—led to a non-differential use of a gaze-centered reference frame. Similar results were obtained by calculating a one-factorial RM-ANOVA (*condition*) on the slopes. The slopes did not differ between conditions (F_1,11_ = 0.04, p = .847) but were significantly different from zero (t > -5.11, p < .001) suggesting a similar influence of gaze in both conditions. Fig 5 shows the results of experiment 2.

The results of experiment 1 implied that the availability of online proprioceptive information about the target location does have a mitigating effect on gaze-centered coding. However, in experiment 2, gaze-dependent reach errors did not vary between the movement-before- and movement-after-target conditions although they differed in the availability of online proprioceptive information about the target. Consequently, either there is a differential effect of the movement before versus after the target presentation concealed by an opposing influence of online information *or* the observed mitigation of the gaze-centered error observed in experiment 1 cannot be attributed to the availability of online information but to another factor.

To elucidate the discrepancy between the results of experiment 1 and 2, we conducted a new condition in which we explored whether target distance from the body rather than the availability of online information led to the apparent influence of proprioceptive online information on gaze-centered coding observed in experiment 1.

#### Exp3: The effect of target distance on gaze-centered coding

We conducted an additional condition (moved-online-far) and compared it with the moved-online and moved-remembered condition of experiment 1. In the moved-online-far condition, participants performed the same task as in the moved-online condition of experiment 1: They moved their left hand to the target location, received a touch on one of two fingertips, moved their hand back, and then reached to the current location of the touched fingertip. However, compared to experiment 1, the apparatus was turned by 180° so that the target locations were now at the side of the apparatus that was closest to the participants and the movement of the left hand started at the rear end of the apparatus. Consequently, when the reach towards the touched finger was performed, the finger was located at the rear end of the apparatus, “far” from the participant, yielding a target distance that was comparable to the target distance of all other conditions except the moved-online condition in which the reach targets were closer to the body. If target distance from the body was the critical factor that diminished the gaze-dependent effects in the moved-online condition of experiment 1, the pattern of reach errors in the moved-online-far condition should be different from the moved-online condition but similar to the moved-remembered condition. Since experiment 3 is exploratory, i.e. the hypotheses were not derived independent from the data but an additional condition (moved-online-far) was performed to be compared with the previously analyzed data, we limit the reports to descriptive statistics. Fig 6 shows how the reach errors vary as a function of gaze relative to target in the moved-online-far condition (red line) and for the same participants’ in the moved-online (-near) and moved-remembered condition (green and yellow line, respectively). The moved-online-far condition shows a considerable overlap with the moved-remembered condition whereas it clearly differs from the moved-online (-near) condition especially when gaze was right of the target.

Thus, the pattern of reach errors between the conditions indicate that—besides a movement of the target limb—target distance influences the use of a gaze-centered reference frame for proprioceptive reach targets.

## Discussion

In a previous study ([10]), we demonstrated that an effector movement before reaching leads to gaze-centered coding of proprioceptive reach targets. Here, we further explored this finding by examining whether the availability of sensory information during the reach, i.e. online versus remembered reaching, also influences the use of a gaze-centered reference frame (Exp1). Moreover, we examined the effect of the time of the effector movement, i.e. whether the movement occurred before versus after target presentation, on gaze-centered coding of proprioceptive reach targets (Exp2). Lastly, based on the results of experiment 1 and experiment 2, we scrutinized the influence of target distance from the body on the use of gaze-centered reference frame (Exp3).

In line with our previous study ([10]), we found that reach errors varied as a function of gaze relative to target in every condition that included a movement of the stimulated hand, but not in the condition in which the stimulated hand remained stationary. This demonstrates that participants, on average, used a gaze-centered reference frame for reaching to a proprioceptive target when they moved the hand that felt the target before the reach. This is likely caused by changes in the weighting of multiple spatial reference frames that are used in parallel ([19]; [8]) and are flexibly adapted to the sensory ([20]), motor ([21]; [10]; [19]), and cognitive context ([22]). In particular, our results indicate that the effector movement increased the weighting of a gaze-centered target representation. This might be beneficial as our visual system, operating in gaze-centered coordinates, is well-trained in rapidly updating locations after eye or body movements in order to maintain spatial stability despite movement ([2]; [23]).

In experiment 1, gaze-dependent errors for reaches to online targets were less pronounced than for reaches to remembered targets. The results of experiment 1 seem to suggest that the availability of online information mitigates the use of a gaze-centered reference frame compared to a situation in which the target has to be remembered in external space. However, the results of experiment 2 questioned this interpretation. In experiment 2, we found a very similar pattern of reach errors when the left hand was moved before versus after target presentation. This result implies a constant updating of the position of the (to be) stimulated hand in gaze-centered coordinates rather than an update of the target location (touch on finger) per se. The movement before target presentation meant that participants had their hand still at the target location when reaching towards it, i.e. online reaching, while the movement after target presentation involved that participants moved their stimulated hand away from the target site before reaching to the location that had to be remembered. Consequently, we would have expected from the results of experiment 1 that there is an effect due to the availability of sensory information about the target location. However, the observation that the gaze-dependent reach errors were similar between the movement-before-target and the movement-after-target condition suggested that either the effect of the movement phases cancels out the effect of the online sensory feedback or, more likely, that the diminished gaze-dependency of reach errors we observed in experiment 1 was not caused by the availability of sensory information, but by other factors. Therefore, we conducted a new condition (moved-online-far condition) to examine whether the shorter target distance from the body in the moved-online condition compared to the other conditions caused the decrease in gaze-centered coding (Exp3). The results of the moved-online-far condition, in which we kept the target distance similar to the other conditions (far reaching), revealed a more pronounced pattern of gaze-dependent reach errors than the moved-online (-near) condition of experiment 1. In fact, the magnitude of gaze-dependent reach errors was similar to the moved-remembered condition of experiment 1 suggesting that, indeed, target distance, even within reachable space, increases the weighting of a gaze-centered representation with a stronger weighting for far than near reach targets. The sagittal distance between the fixation sites and the target locations was kept similar in the moved-online (-near) and—far condition; thus, the observed differences in reach errors cannot be attributed to the fixation-target distance but depend on the target distance relative to the body.

Comparing the two moved-online conditions (near versus far) that differ with respect to target distance, reaches to the far location tended to be more variable than reaches closer to the body. One might argue that the more pronounced pattern of gaze-dependent reach errors might thus depend on participants making larger errors because of the larger distance instead of reflecting a change in the weighting of reference frames. However, we observed a pattern of reach errors that specifically varied with gaze direction rather than a general increase of errors independent of gaze. Overall, we interpret the size (or the slope) of the gaze-dependent reach errors as a measure for the influence contribution of a gaze-centered reference frame. While some studies investigating reaching to proprioceptive targets using other measures than gaze-dependent reach errors support some of our results ([20]: gaze-independent coding of stationary targets; [19]: transition from gaze-independent to gaze-centered coding when stimulated limb is moved compared to stationary), we cannot exclude the possibility that the eccentricity-dependency of the error changes independently from the reference frame that is used.

Our results further showed that participants reached more precisely in the moved-online conditions (near and far) than in the moved-remembered condition which is in line with studies on the use of reference frames for visual reach targets ([24]; [15]). They found that memory delays increased the variability of reach errors but not their overall pattern promoting gaze-centered coding. This indicates that while the reliability of sensory information is affected by the delay, the coding scheme is not. This is supported by the results of experiment 1 showing that online versus remembered proprioceptive reaching had no differential influence on the gaze-centered coding scheme, as has also been demonstrated for immediate versus memory-guided reaching to visual targets ([24]; [15]).

In sum, the results of the present study suggest that the observed gaze-dependent effects on proprioceptive reaching are due to the effector movement rather than the availability of online information about the reach target. Thus, an effector movement leads to gaze-centered coding of both online and remembered proprioceptive reach targets. Such gaze-centered coding seems to be used to constantly update the positional signals of the hand on which the target is applied rather than the target location per se. Moreover, target distance influences gaze-centered coding such that more weight is assigned to the gaze-centered representation when reaching to targets farther away than close to the body and to the starting position respectively.
